# Free Tools and Strategies for the Generation of 3D Finite Element Meshes: Modeling of the Cardiac Structures

**DOI:** 10.1155/2013/540571

**Published:** 2013-05-16

**Authors:** E. Pavarino, L. A. Neves, J. M. Machado, M. F. de Godoy, Y. Shiyou, J. C. Momente, G. F. D. Zafalon, A. R. Pinto, C. R. Valêncio

**Affiliations:** ^1^Department of Computer Science and Statistics (DCCE), São Paulo State University (UNESP), 15054-000 São José do Rio Preto, SP, Brazil; ^2^Department of Cardiology and Cardiovascular Surgery, São José do Rio Preto Medical School–Famerp, 15090-000 São José do Rio Preto, SP, Brazil; ^3^College of Electrical Engineering, Zhejiang University, Hangzhou 310027, China

## Abstract

The Finite Element Method is a well-known technique, being extensively applied in different areas. Studies using the Finite Element Method (FEM) are targeted to improve cardiac ablation procedures. For such simulations, the finite element meshes should consider the size and histological features of the target structures. However, it is possible to verify that some methods or tools used to generate meshes of human body structures are still limited, due to nondetailed models, nontrivial preprocessing, or mainly limitation in the use condition. In this paper, alternatives are demonstrated to solid modeling and automatic generation of highly refined tetrahedral meshes, with quality compatible with other studies focused on mesh generation. The innovations presented here are strategies to integrate Open Source Software (OSS). The chosen techniques and strategies are presented and discussed, considering cardiac structures as a first application context.

## 1. Introduction

 The increased use of minimally invasive surgical procedures in medicine is a reality, with applications in different specialties. The small incisions ensure the patient smaller exposure to infections, as well as a quicker recovery. The radiofrequency cardiac ablation is a good example of it, being extensively used for over 10 years in the treatment of tachycardia, atrial fibrillation, and atrial flutter [1–4]. This technique is not free from complications, although it has advanced in the last decade. The esophageal injury is a common damage, characterized by the union of tissues from the left atrium and esophagus, through necrosis [1, 4]. The consequence for the patient is death caused by internal bleeding, as blood is diverted directly to the stomach, when it is not noticed by the physician.

In the literature, studies using the Finite Element Method (FEM) are targeted to improve cardiac ablation procedure and reduce possible complications, such as esophageal injury. It is possible to highlight that the nucleus of the problem is monitoring the temperatures in the tissues involved more accurately. This approach is not simple, and the computational simulation using FEM has contributed significantly to the improvement of this technique [5–12]. For such simulations, the finite element meshes should consider the size and histological features of the target structures. Furthermore, the quality of the meshes is another fundamental property to properly simulate the desired phenomena. The techniques which are able to generate meshes with such characteristics are preferred, and when they are generated with open source codes, they make easier tests with no user restrictions. These properties can guarantee more accurate and clinically relevant simulations.

In this context, it is possible to verify that some methods or tools used to generate meshes of human body structures are still limited by providing or using nondetailed models [5–8, 10, 13–15], by the need of nontrivial preprocessing, which is a primary step applied to define, extract, or change the anatomical features (boundary domain) required by meshing generation step [7, 11, 15–18] or due to limitations of the user condition [5, 6, 8, 12, 13, 18, 19]. One of the reasons for this finding is the necessary commitment to represent the complicated geometries of the involved domains, which requires sophisticated resources being sometimes under development in specific tools for geometric modeling and mesh discretization [20, 21]. A typical integrated software tool to construct three-dimensional domains and finite element meshes may have a development time cycle of more than 10 years [22, 23] and frequently with exceptions to allow linking with other mesh generators. An alternative is integrating Open Source Software packages dedicated to solid modeling with automatic generation of tetrahedral meshes, which are available in the literature. This strategy brings obvious advantages in the context of FEM simulations.

With these findings, the present paper demonstrates alternatives to solid modeling and automatic generation of highly refined tetrahedral meshes and with quality compatible with other studies focused on mesh generation. The innovations presented here are strategies to integrate Open Source Software (OSS). The chosen tools were the Blender software [24] as solid modeler and the TetGen as automatic mesh generator [25], which uses the Delaunay tetrahedralization [25, 26]. Furthermore, in this study we demonstrate cardiac structures as a first application context, motivated by the importance that the meshes of these structures represent for studies of cardiac ablation. In the next sections a discussion concerning our strategy for software integration and performance tests in realistic application domain are presented.

## 2. Material and Methods

 The proposed methodology for solid modeling and automatic generation of tetrahedral meshes was organized in Section 2.1, with details about the defined anatomical properties for the application context; Section 2.2, with definitions of the used packages and corresponding justifications; Section 2.3, with details about the recommendations to discretize domains; and Section 2.4, with specifications from the integration process of the models built on the solid modeler to the algorithm used in the automatic mesh generator. The proposal will be described in detail in the next subsections.

### 2.1. Application: Features of Cardiac Structures

**Figure 1:**
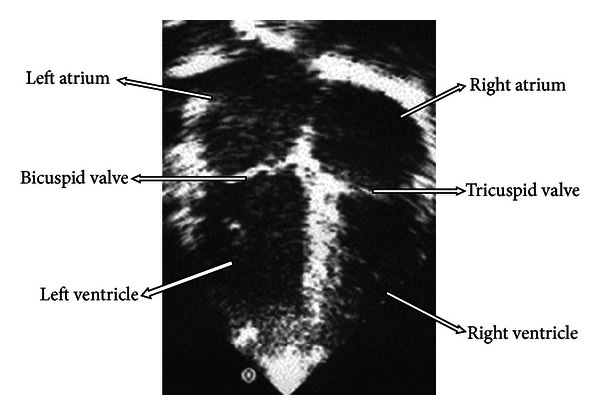
Example of echocardiography used in the modeling process. The aortic valve and the pulmonary valve do not appear in this figure.

 The choices of cardiac structures were motivated by the complicated geometric domain and by the clinical relevance that these structures represent for the investigation of esophageal injury. Therefore, the model defined in our study was composed of cardiac regions consisting of two main parts: the right portion (venous) and the left portion (arterial). Each portion has an atrium, a ventricle, and valves: bicuspid, and tricuspid, pulmonary or aortic valve. The trunks of aorta and pulmonary artery were represented from their connections with the atriums to the beginning of their ramifications. The dimensions of these structures are presented in Table 1 [27–29], defined in cardiac diastole (period of heart muscle relaxation) and values present in major axes. The presence of some structures is visualized on an echocardiographic image (Figure 1), which was used as another reference during the modeling process. 

### 2.2. Free and Open Source Packages

 Creating complex three-dimensional models is not a trivial task, especially without the support of sophisticated modelers and already equipped with resources to integrate it with algorithms responsible for mesh generation. The Blender package [24] maintained by Blender Foundation was chosen. This package is an integrated system of tools, a multiplatform and contains resources to export and import objects in different formats, through scripts. Scripts are useful for automating methods, navigating and manipulating the discretized geometric domain. Moreover, Blender is available under a dual license, Blender License (BL) and GNU General Public License. With all these resources, it becomes possible to represent the domain of interest and use scripts, written in Python language, to export features required by the automatic mesh generator.

The stage for automatic generation of tetrahedral meshes is not a trivial task, a fact that limits the use of a single algorithm to discretize the most different contexts. The algorithms commonly applied to generate tetrahedral meshes have good and bad points [26, 30–32], some of which require manual interference in the domain to obtain the desired discretization. Although there are different methods for generating three-dimensional meshes, we chose the Delaunay algorithm [25, 31] for being one of the most popular and one of the most efficient algorithms [30], available in the TetGen package [26]. Just as Blender, TetGen is an Open Source Software (OSS) and is available under MIT License. This package is maintained by the research group called Numerical Mathematics and Scientific Computing, Weierstrass Institute for Applied Analysis and Stochastics (WIAS), Berlin, Germany.

The selected packages for the models construction and automatic generation of meshes were explored in a computer with a 2.40 GHz quad core processor and 16 GB of RAM memory. The operating system used uses 64-bit architecture, running Blender in version 2.49b and TetGen mesh generator in version 1.4.

### 2.3. Definition of Strategies for Solid Construction

 The representation of solids in the Blender package must respect two strategies to ensure the integration with the automatic mesh generator. The strategies or recommendations are (1) definitions of faces and (2) density control of vertices, whereas the application of each must consider the complexity of the specific region.

**Figure 2:**
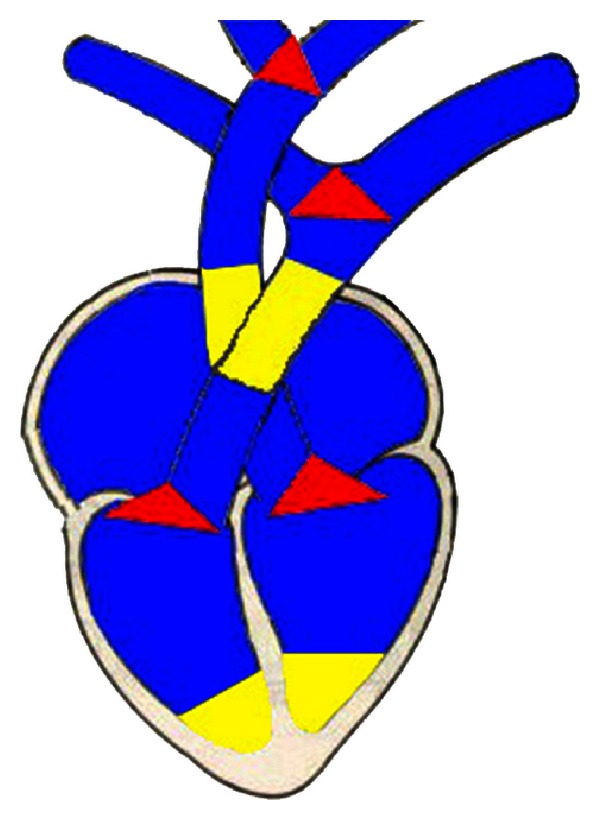
Chosen regions for quadrilateral or rectangular faces (blue), triangular faces (red), and with increased density of vertices (yellow).

The strategy definitions of faces consist of choosing the most appropriate types of faces to discretize a solid. Regular or noncomplex regions of the specific domain must be discretized with quadrilateral or rectangular faces. A quadrilateral or rectangular face can be modified to fill regions with sharp angles, since the values of the internal angles of the faces are between 30 and 160 degrees. The limits were defined on the basis of the stage of mesh generation, according to the values which propitiated intact and highly refined meshes. In the application focused on this study, quadrilateral or rectangular faces were used to discretize the atriums, the ventricles, the trunks of aorta and pulmonary artery, and the bicuspid, tricuspid, aortic, and pulmonary valves, which are regular and cylindrical regions (marked in blue in Figure 2). Regions with triangular faces were constructed where the discretization required faces with internal angles out of the predetermined range.

The triangular faces are equilateral, isosceles or scalene elements and allow more appropriated representations of regions with sharp angles, such as those occurring at bifurcations points. In the application explored in this study, that situation is commonly evidenced in the bifurcations of arteries and veins, as well as in the regions of connection between atriums, ventricles, and arteries. These regions were demarcated in red in Figure 2.

The strategy density control of vertices defines the number of nodes in a region. The increasing number of vertices allows a better representation of curved regions, by smoothing the direction transition and respecting the features defined on the first property and the orthogonality of the faces (Figure 2). In the context of cardiac structures, this strategy was applied in the construction of cardiac valves, bases of ventricles and in the change of the direction of the aorta and pulmonary artery responsible for their correct positions.

**Figure 3:**
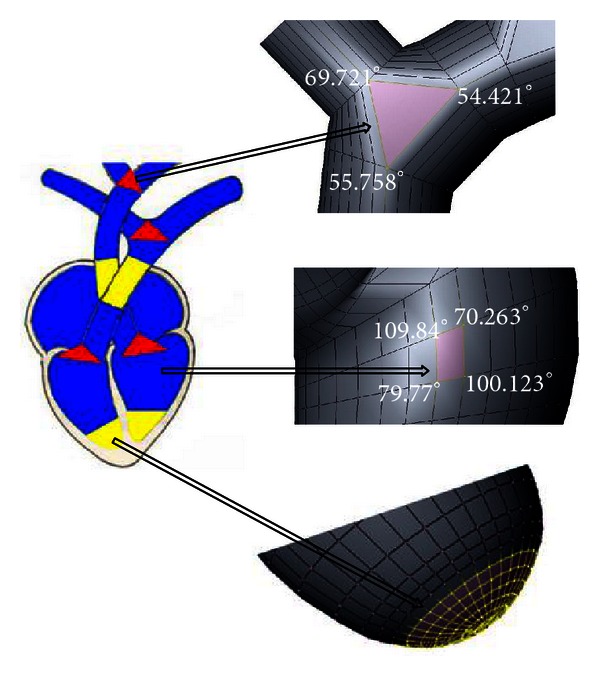
Example of regions represented with triangular faces, quadrilateral or rectangular faces, and with increased density of vertices.

Figure 3 shows examples of regions generated with the strategies or recommendations described previously. 

### 2.4. Application of Integration Strategies

**Figure 4:**
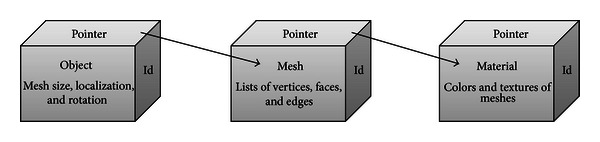
Illustration of objects, meshes, and materials data block structures in a “blend file”.

**Figure 5:**
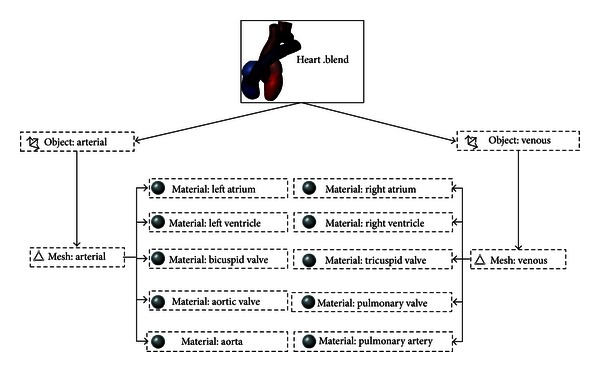
Hierarchy of the heart model determined by Blender, considering object, mesh, and material data blocks.

 A domain discretized in Blender is stored in its native file format: a “blend file”. The data used to discretize a solid are stored in this standard file in structures named data blocks. Data blocks are data structures similar to the heterogeneous structure type. In a simple domain, several data blocks may be used. The data blocks can be made of object, mesh, material or scene type, including many others. A data block made of object type stores information about the mesh size and the linear transformations applied; a datablock made of mesh type stores information about the vertices, edges, and faces of the model; a datablock made of material type stores information about the colors assigned to a certain set of vertices, edges or faces. These colors are also called markers. Therefore, the datablocks are linked by pointers and defines a data structure of tree type (Figure 4).  Figure 5 exemplifies the structures described for material, mesh, and object datablocks used to represent each cardiac structure, based on Blender Architecture, requiring a total of 14 datablocks. 

**Figure 6:**
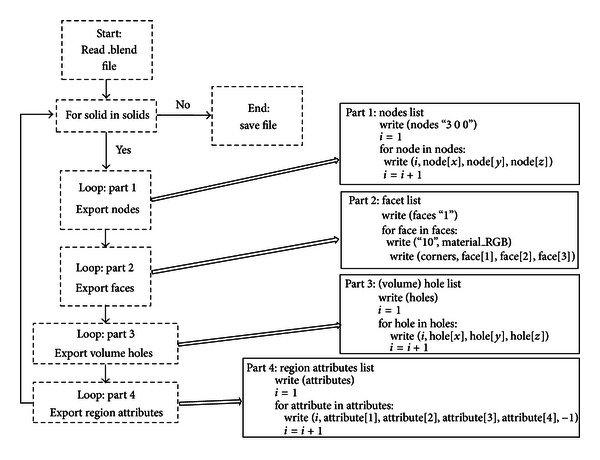
Proposed method to repeat automatically the process for the group of existing solids and generate a single ASCII file.

The integration of the models constructed on Blender with the mesh generator TetGen was accomplished by Python script [32]. The script operates into objects generated by the Blender package through four iterative structures. These structures read the data contained in mesh and object data blocks and translate the information about vertices, faces, holes, and attributes accordingly. The processed information is an ASCII file (.poly), properly written in the formats required by TetGen. The Python script presented by [32] exports only the active solid, which limits the potential for the meshing of mixed solids. The modification performed repeats the process for the group of existing solids automatically and generates a single ASCII file (.poly).  Figure 6 shows a diagram of the algorithm used for exportation, with the proposed modification.

A typical “poly file” is composed by 4 parts: an indexed list of point coordinates; a list of solid faces; a list of volume holes; and a list of attributes or boundaries (constraints), see Supplementary Material (Appendix 1) available online at http://dx.doi.org/10.1155/2013/540571.

**Figure 7:**
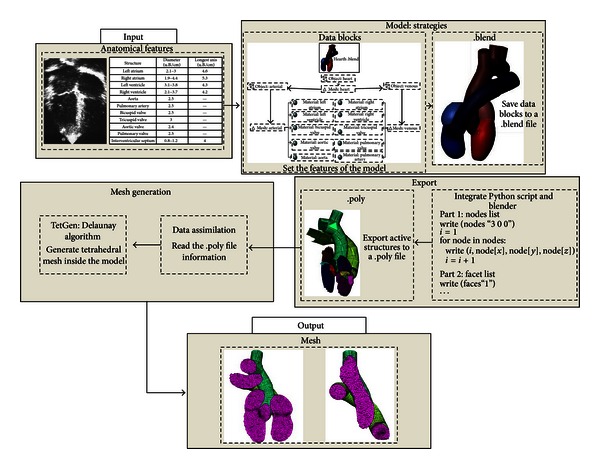
Flowchart of the steps of integration between the tools used.

Figure 7 represents the integration strategies of the Blender and TetGen using the Python script (Figure 6).

This process generates three files. A “node file” contains a list of three-dimensional points. Each point has three coordinates (*x*, *y*, and *z*) and probably includes one or several attributes and a boundary marker as well. A “ele file” contains a list of tetrahedrons. Each tetrahedron has four corners. Nodes are indices into the corresponding “node file”. The four nodes are the corner vertices. A “face file” contains a list of triangular faces, which may be boundary faces, or convex hull faces. Each face has three corners and possibly a boundary marker. Nodes are indices into the corresponding “node file” [25].

## 3. Results and Discussions

**Figure 8:**
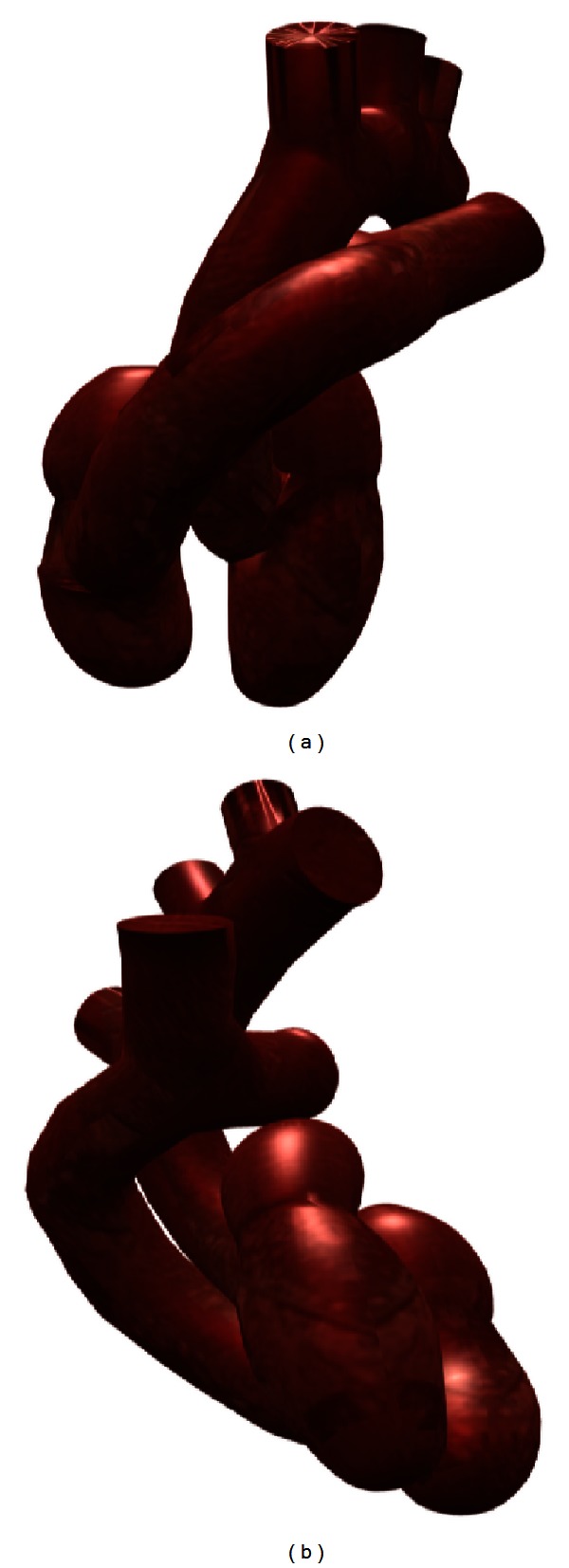
Model constructed with the approaches previously presented, of which an anterior view is in (a) and a lateral view is in (b).

 In this work, the cardiac structures were chosen as a first application context, since three-dimensional meshes of these structures are relevant for studies of cardiac ablation. The complicated geometric domain selected allowed to test the feasibility of the combined use of anatomical features (Section 2.1), free and Open Source Packages (Section 2.2) and apply strategies (Sections 2.3 and 2.4). The organization of the cardiac structures was represented in Blender, considering atriums, ventricles, valves (bicuspid, tricuspid, pulmonary, and aortic), and artery trunks (aorta and pulmonary artery). The model is shown in Figure 8, in different projections. 

**Figure 9:**
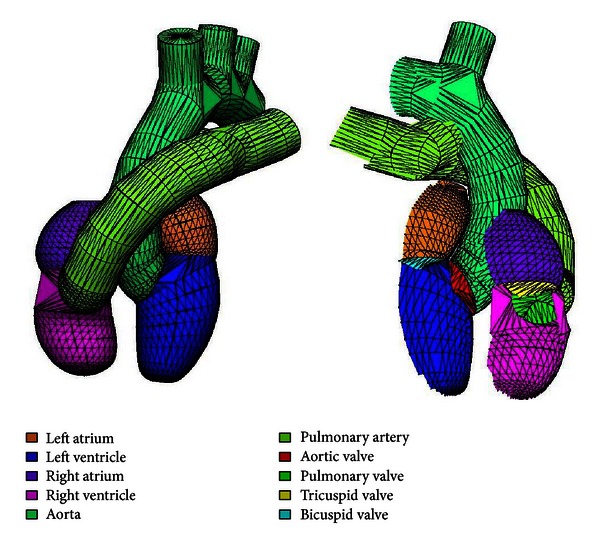
Structures with their corresponding boundary markers.

The “poly file” obtained from the strategies described above is partially shown in Supplementary Material (Appendix 2). The file stores data of 3818 vertices, 3980 faces and 10 boundary markers. Boundary markers are numerical codes such as −1000000 used to assign the blue color to the faces of left ventricle. Different colors are used to better distinguish each structure. Boundary markers are normally used to simulations in specific regions. A preview of each structure and its boundary markers is represented in Figure 9. 

**Figure 10:**
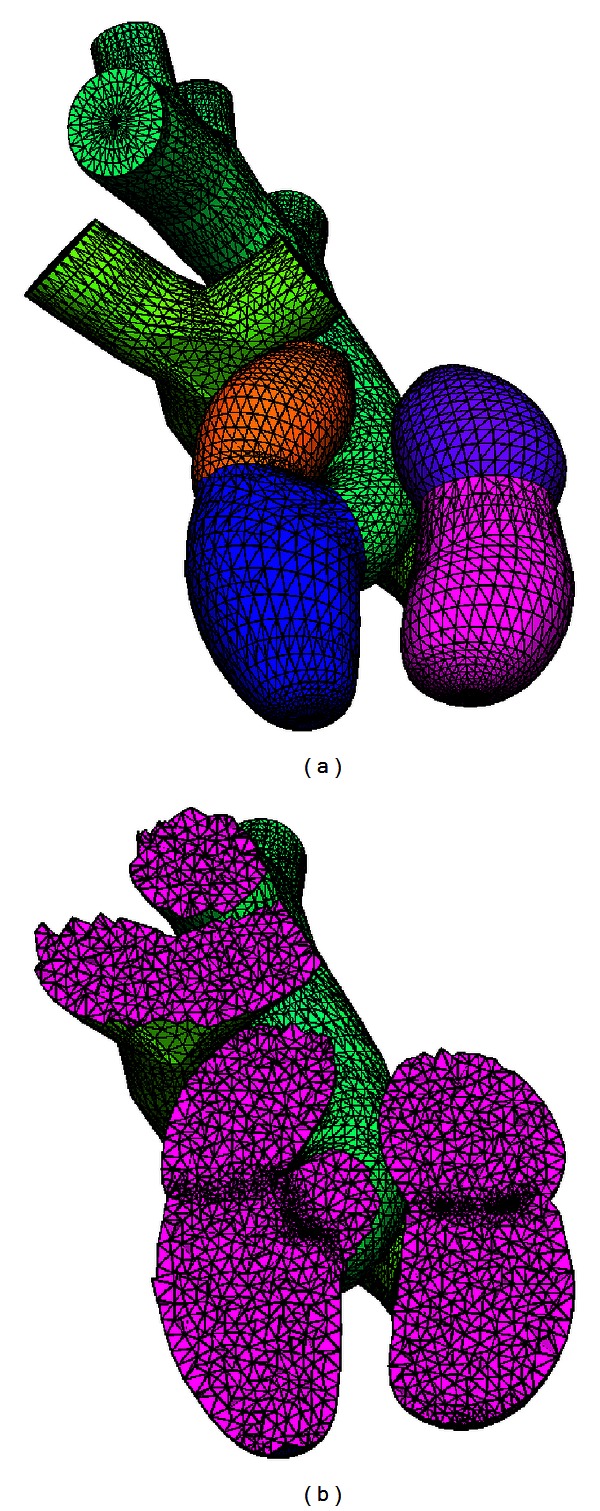
A raw mesh is shown in (a), and an *x*, *y* plane cut is shown in (b).

A raw mesh was generated from the proposal presented, and a cut was made to better illustrate the quality of the mesh.  Figure 10 shows a mesh constructed with 33875 faces, 42371 nodes, 10 boundary markers, and 223851 tetrahedral elements, a useful example to validate the concept of iteration loop described in the previous section.

**Figure 11:**
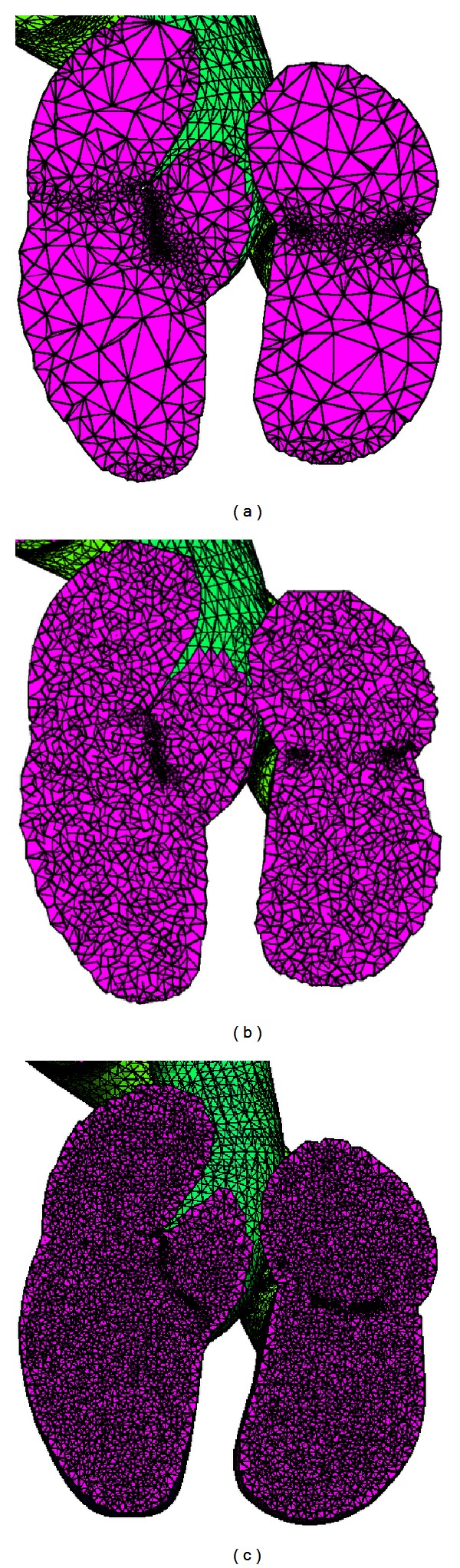
Regions of the atriums and ventricles to exemplify the increasing number of nodes in each refinement performed. The number of tetrahedral elements in each test was (a) 158610, (b) 451764, and (c) 1956490.

**Figure 12:**
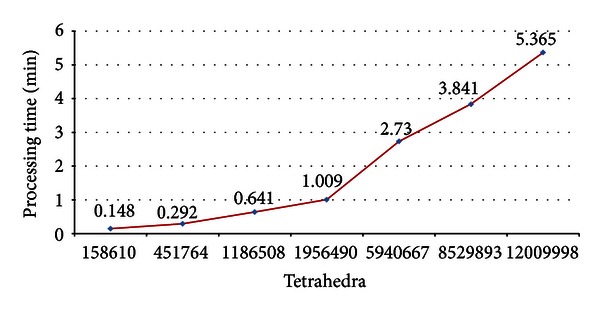
Meshing time (*y*-axis) as a function of the number of elements in the tetrahedral mesh (*x*-axis), for each refinement shown in Figure 11.

Studies grounded in FEM require tools with resources to generate highly refined meshes of simple or complex domains, and in an acceptable time. The methodology proposed in this paper meets these requirements. For the chosen complicated application context, meshes were generated through successive refinements of the coarse initial mesh (Figure 10). Regions of the atriums and ventricles were used to demonstrate the number of tetrahedral elements presented in some refinements (Figure 11). It was therefore possible to estimate the processing time growth, and the number of tetrahedral elements increased. These results are shown in Figure 12 for each test performed. Just a few studies show how many elements have their meshes [16, 19]. Also, none of these studies provide information about the meshing time. This is a limitation for comparisons of our results. 

The mesh quality is another important aspect that should be considered in proposals designed to generate meshes through FEM simulations. The number of meshes elements can be an important criterion for this. Another criterion commonly adopted is showing the dihedral angles obtained. The quality of a tetrahedral element is commonly measured in terms of minimal and/or maximal dihedral angles. The success of the Finite Element Method depends on the shapes of these tetrahedra. For instance, tetrahedra constructed with too small or too large dihedral angles can cause interpolation errors and lead to numerical simulation with higher instability and less accuracy [33, 34]. The desired quality is the one in which the values of dihedral angles are close to the values set on a regular tetrahedron [26, 34, 35]. This can be verified in histograms constructed with the total of dihedral angles present in ranges of angles, as well as identification of the smallest and largest values involved [33, 34].

**Figure 13:**
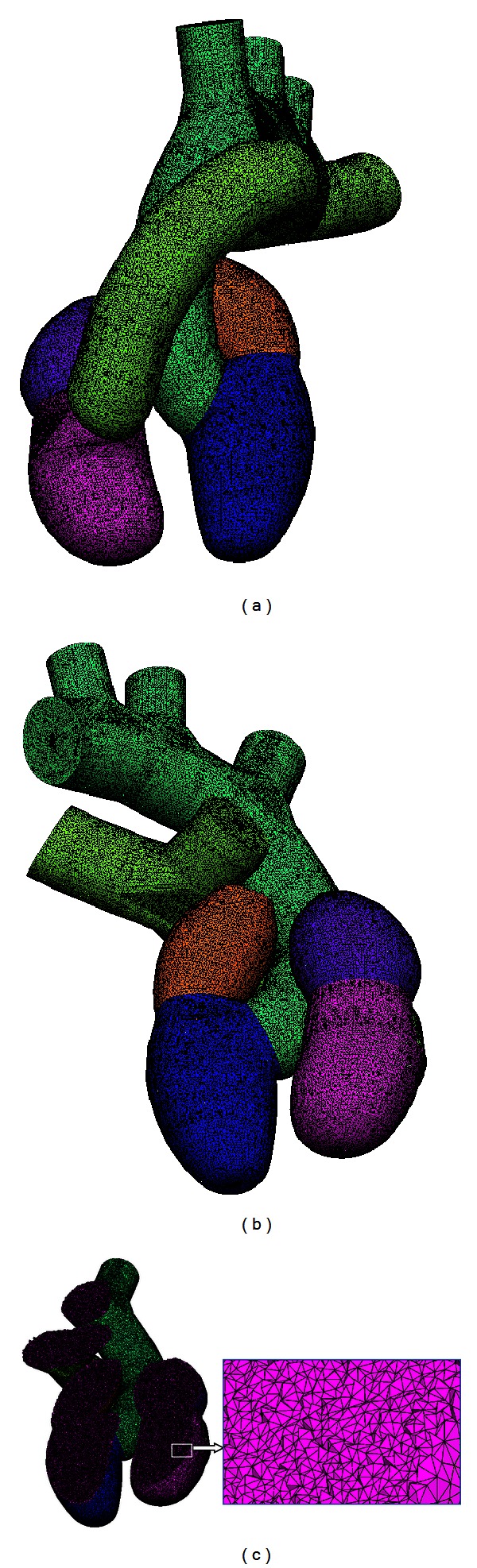
Mesh constructed with 12009998 tetrahedra in 5.36 minutes is shown in anterior (a) and posterior (b) views. A zoom view detail is given in (c), according to a transversal cut applied in the plane *x*, *y*.

**Figure 14:**
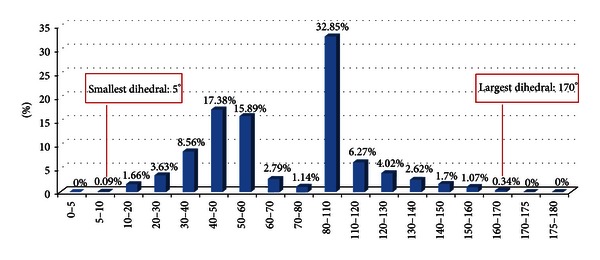
Histogram constructed with the total of dihedral angles (*y*-axis) inside prescribed angle intervals in degrees (*x*-axis). The corresponding tetrahedral mesh was shown in Figure 13.

It is possible to verify through the presentation of the mesh constructed with 12009998 tetrahedral elements (Figure 13) and the corresponding histogram (Figure 14) that our proposal allows obtaining highly refined meshes from complex domains, in some acceptable time and quality. This number of elements may facilitate the representation of degenerated tetrahedral elements and effectively indicate the limits of the proposed approach. However, it is also possible to verify that the tetrahedral elements have dihedral angles whose values belong to the interval which varies from 5 to 170 degrees, with peak values around the condition for a regular tetrahedron. These values are close to those used to determine the mesh quality in other studies [33, 34]. For instance, the algorithm proposed by [34] is capable of producing tetrahedral meshes whose dihedral angles are bounded between 10.7 and 164.8 degrees or between 8.9 and 158.8 degrees with a change in parameters. Also, when nonuniform tetrahedra on the surface boundary are chosen, the dihedral angles are bounded between 1.66 and 174.72 degrees. In another example, tetrahedral meshes are generated with the minimal dihedral angle being guaranteed to be greater than or equal to 5.71 degrees [33].

## 4. Conclusion

 The proposal presented in this paper considered strategies to build solid and automatic mesh generation, based on Open Source Packages. The strategies are feasible to generate highly refined mesh in some acceptable time and with the required quality for simulation using the Finite Element Method (FEM). These facts were demonstrated by considering a complex domain with some practical importance, as in the case of mesh structures for the study of cardiac ablation through FEM. The most refined mesh involved approximately 12 million elements and was generated in 5.35 minutes. Despite the significant number of tetrahedral elements involved, the explored example does not define the limit of possible refinements from our approach. The tetrahedral quality was also discussed and the values of dihedral angles generated are consistent with the literature. So, our proposal provides a significant contribution to the mesh generation for studies using FEM, which is a known method applied in different areas.

## Supplementary Material

Appendix 1: shows the file structure required as input by the TetGen package.
Appendix 2: shows the structure of the “poly file” for the cardiac structures.

## Figures and Tables

**Table 1:** Values used to define the heart model. Considering the diastolic dimensions of an adult heart [27–29].

Structure	Dimension (cm)	Longest axis (cm)
Left atrium	2.1–3.0	4.6
Right atrium	1.9–4.4	5.3
Left ventricle	3.1–3.8	4.3
Right ventricle	2.1–3.7	4.2
Aorta	2.5	—
Pulmonary artery	2.5	—
Bicuspid valve	2.5	—
Tricuspid valve	3.0	—
Aortic valve	2.4	—
Pulmonary valve	2.3	—
Interventricular septum	0.8–1.2	4.0
