# Supplementary material for: Free Tools and Strategies for the Generation of 3D Finite Element Meshes: Modeling of the Cardiac Structures
Source: Int J Biomed Imaging. 2013 May 16;2013:540571. doi: 10.1155/2013/540571 (PMC3670575; doi:10.1155/2013/540571)
Supplement: Supplementary file 1 — Appendix 1: shows the file structure required as input by the TetGen package. Appendix 2: shows the structure of the “poly file” for the cardiac structures. [file 540571.f1.pdf]

Free Tools and Strategies for the Generation of 3D Finite Element Meshes: Modeling of the Cardiac Structures

---

Supplementary Material

1. Appendix 1: File structure required as input by the TetGen package [1].

Part 1 - node list

```
First line:  <# of points> <dimension (3)> <# of attributes>
             <boundary markers (0 or 1)>
Remaining lines list # of points:
  <point #> <x> <y> <z> [attributes] [boundary marker]
  ...
```

Part 2 - facet list

```
One line: <# of facets> <boundary markers (0 or 1)>
Following lines list # of facets:
   <facet #>
   ...
```

Part 3 - (volume) hole list

```
One line: <# of holes>
Following lines list # of holes:
  <hole #> <x> <y> <z>
  ...
```

Part 4 - region attributes list

```
One line: <# of region>
Following lines list # of region attributes:
  <region #> <x> <y> <z> <region number> <region attribute>
  ...
```

Preprint submitted to International Journal of Biomedical Imaging *April 9, 2013*

2. Appendix 2: The structure of .poly file for the cardiac structures.

```
#Boundary markers
# -61089 Material: Right Ventricle
# -335400 Material: Right Atrium
# -1000000 Material: Left Ventricle
# -934747 Material: Left Atrium
# -68914 Material: Bicuspid Valve
# -1009820 Material: Aortic Valve
# -451414 Material: Aorta
# -1000050 Material: Pulmonary Valve
# -50233 Material: Pulmonary Artery
# -68965 Material: Tricuspid Valve
# Part 1 - node list
# node count, 3 dim, no attribute, no boundary marker
3818 3 0 0
# Node index, node coordinates
1       3.611 -0.458 5.338
2       3.74 -0.287 5.36
3       3.836 -0.094 5.364
4       3.897 0.112 5.351
.
.
.
3817    3.293 -0.889 1.429
3818    3.276 -0.932 1.14
# Part 2 - facet list
# Facet count
3980    1
# facets
1       0 -934747       #1 polygon, no hole, boundary marker (Left Atrium)
4       1 33 34 2
.
.
.
1       0 -1000000      #1 polygon, no hole, boundary marker (Left Ventricle)
4       575 576 577 578
.
.
.
4       1058 1059 1091 1090 #1 polygon, no hole, boundary marker (Aorta)
1       0 -451414
.
.
.
# Part 3 - hole list
0       # no hole
# Part 4 - region list
0       # no region
```

References

[1] H. Si, A. TetGen, A quality tetrahedral mesh generator and three-dimensional delaunay triangulator, Weierstrass Institute for Applied Analysis and Stochastic, Berlin, Germany.
